# Automated Synthesis of ^18^F-BCPP-EF {2-*tert*-Butyl-4-Chloro-5-{6-[2-(2[^18^F]fluoroethoxy)-Ethoxy]-Pyridin-3-ylmethoxy}-2*H*-Pyridazin-3-One for Imaging of Mitochondrial Complex 1 in Parkinson’s Disease

**DOI:** 10.3389/fchem.2022.878835

**Published:** 2022-03-30

**Authors:** Tanpreet Kaur, Allen F. Brooks, Katherine M. Liddell, Bradford D. Henderson, Brian G. Hockley, Nicolaas I. Bohnen, Roger L. Albin, Peter J. H. Scott

**Affiliations:** ^1^ Department of Radiology, The University of Michigan Medical School, Ann Arbor, MI, United States; ^2^ Department of Neurology, University of Michigan, Ann Arbor, MI, United States; ^3^ Neurology Service and Geriatrics Research, Education, and Clinical Center, Veterans Affairs Ann Arbor Healthcare System, Ann Arbor, MI, United States; ^4^ University of Michigan Udall Center of Excellence for Parkinson’s Disease Research, Ann Arbor, MI, United States

**Keywords:** MC-1 inhibitors, BCPP-EF, fluorine-18, green chemistry, radiochemistry, positron emission tomography

## Abstract

Mitochondrial complex I (MC-I) is an essential component of brain bioenergetics and can be quantified and studied using positron emission tomography (PET). A specific high affinity ^18^F radiotracer for MC-I enables monitoring of neurodegenerative disease progression and pathology *via* PET imaging. To facilitate clinical research studies tracking MC-I activity in Parkinson’s disease and other neurodegenerative diseases, a fully automated synthesis of the recently described 2*-tert*-butyl-4-chloro-5-{6-[2-(2[^18^F]fluoroethoxy)-ethoxy]-pyridin-3-ylmethoxy}-2*H*-pyridazin-3-one ([^18^F] BCPP-EF, **[** ^**18**^ **F]1**) was developed. We report the first automated synthesis [^18^F]BCPP-EF using a green radiochemistry approach. The radiotracer was synthesized with good radiochemical yield, excellent radiochemical purity, and high molar activity.

## Graphical Abstract

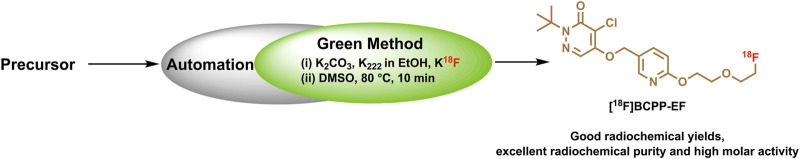

## 1 Introduction

**FIGURE 1:**
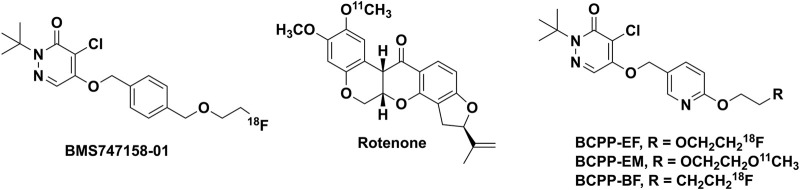
Reported MC-1 inhibitors for mitochondrial imaging.

In eukaryotic cells, mitochondria are crucial organelles for oxidative metabolism and adenosine triphosphate (ATP) production (Kadenbach, 2012). Mitochondrial respiratory chain complexes (I-V) of the inner membrane of mitochondria mediate a cascade of electron transfer for oxidative phosphorylation (OXPHOS) and aerobic adenosine triphosphate (ATP) (Kadenbach, 2012). The largest complex of the electron transport chain (ETC) is mitochondrial respiratory chain complex 1 (MC-1; NADH-ubiquinone oxidoreductase, EC 1.6.5.3), which catalyzes electron transfer from NADH to ubiquinone, translocating four protons through complexes I-V (Parey et al., 2020). MC-1 plays a crucial role in mitochondrial function (Tsukada et al., 2014b) and its dysfunction is linked to physiological brain aging (Tsukada et al., 2014b), kidney malfunctions (Saeki et al., 2020), and ischemic stroke (Suzuki et al., 2021). Human post-mortem and animal model data link MC-1 dysfunction to Alzheimer’s and Parkinson’s diseases (Tsukada, 2016; Kanazawa et al., 2017; Barron et al., 2020; Fang et al., 2020) Even a small loss of MC-1 activity significantly affects ATP synthesis and mitochondrial respiration in brain mitochondria (Pollard et al., 2016; Wilson et al., 2020). For better understanding of the metabolic role of MC-1 in the pathology of various diseases, positron emission tomography (PET) imaging of MC-1 is of interest and several MC-1 imaging agents are reported (Figure 1).

The University of Michigan PET Center conducted work with ^11^C-labeled derivatives of rotenone in the past (Charalambous et al., 1995a,b; Kilbourn et al., 1997; Snyder et al., 1999), but these agents were not widely used, likely because of toxicity considerations. More recently, a pyridazinone analog, (^18^F)BMS-747158-01 (Flurpiridaz), which advanced to clinical trials as a myocardial perfusion imaging agent (Sattler et al., 2014), showed inhibitory activity for MC-1 function (Rokugawa et al., 2017). Studies with the agent demonstrated high uptake and long retention not only in the heart (Werner et al., 2019), but also in the brain. (^18^F)BMS-747158-01, however, exhibited high non-specific binding both *in vivo* and *in vitro* in the brain compared with the specific MC-1 known inhibitor rotenone (Maddahi et al., 2011).

**FIGURE 2:**
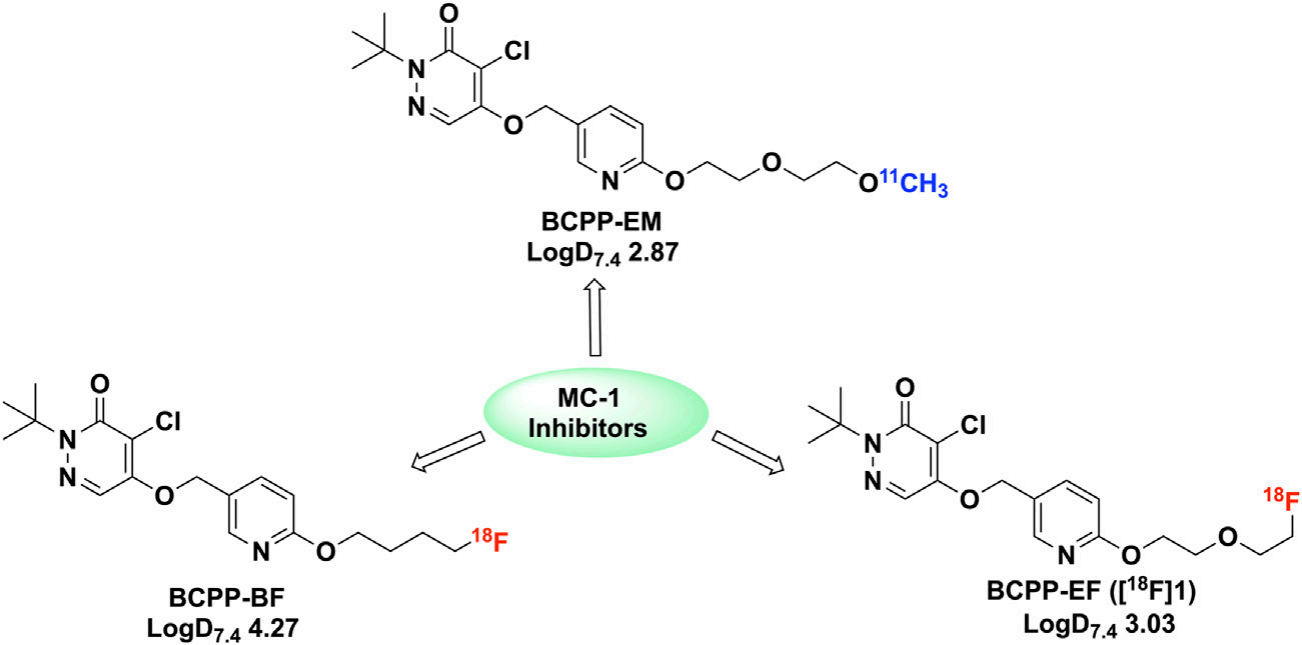
^18^F and ^11^C labelled BCPP analogues (BCPP-EM, BCPP-EF and BCPP-BF) studied for PET imaging of MC-1 inhibitors (Harada et al., 2013).

Further work aimed at tuning the CNS imaging properties led to three additional analogues: BCPP-EF, -BF, and -EM (Figure 2) (Harada et al., 2013) Our interest was directed towards BCPP-EF and -BF, given the use of fluorine-18 (t_1/2_ = 109.8 min) versus carbon-11 (t_1/2_ = 20.4 min) for radiolabelling. Labelling with fluorine-18 offers better signal to background, potential additionial time points for study with the longer half-life, and easier distribution to off-site PET imaging facilities. Another issue associated with BCPP-EM was radiolysis at high molar activity (332 ± 228.5 GBq/μmol), which resulted in low radiochemical purity (Harada et al., 2013). The highly lipophilic nature of BCPP-BF (logD_7.4_ 4.2) resulted in high non-specific binding (34%), and increased bone uptake was observed with the agent (Harada et al., 2013). Based on these previous studies, it was determined that BCPP-EF (**1**) demonstrated promising quantitative imaging of MC-I activity and its ischemic damage in the living brain with PET without confounding microglial activation (Tsukada et al., 2014a). BCPP-EF exhibited greater sensitivity towards rotenone and lower non-specific binding (4%) (Harada et al., 2013). We decided to synthesize BCPP-EF and fully automate the method for clinical use at our PET Center. We report the first automated synthesis of (^18^F)BCPP-EF using a green radiochemistry approach (Shao et al., 2014; Stewart et al., 2015) that provides radiotracer with good radiochemical yield, excellent radiochemical purity, and high molar activity. We also provide quality control testing results that confirm the product is suitable for clinical use.

## 2 Materials and Methods

### 2.1 Materials and Methods

#### 2.1.1 Materials

Reagents and solvents were purchased from Aldrich Chemical or Fisher Scientific and were used without further purification unless noted. Chromatography columns for HPLC analysis and purification were purchased from Phenomenex or Waters. High performance liquid chromatography (HPLC) was performed using a Shimadzu LC-2010A HT system equipped with a Bioscan B- FC-1000 radiation detector. Sodium chloride, 0.9% USP and sterile water for Injection, USP were purchased from Hospira; Dehydrated Alcohol for Injection, USP was obtained from Akorn Inc. Sterile filters were acquired from Millipore; 10 cc sterile vials were obtained from Hollister-Stier; C18 sep-paks were purchased from Waters Corporation and were flushed with 10 ml of ethanol followed by 10 ml of sterile water prior to use. BCPP-EF reference standard **1** and labeling precursor **15** were synthesized in house. Detailed procedures are provided in the Supplementary Material.

#### 2.1.2 Procedure for Radiochemical Synthesis of [^18^F]1

**FIGURE 3:**
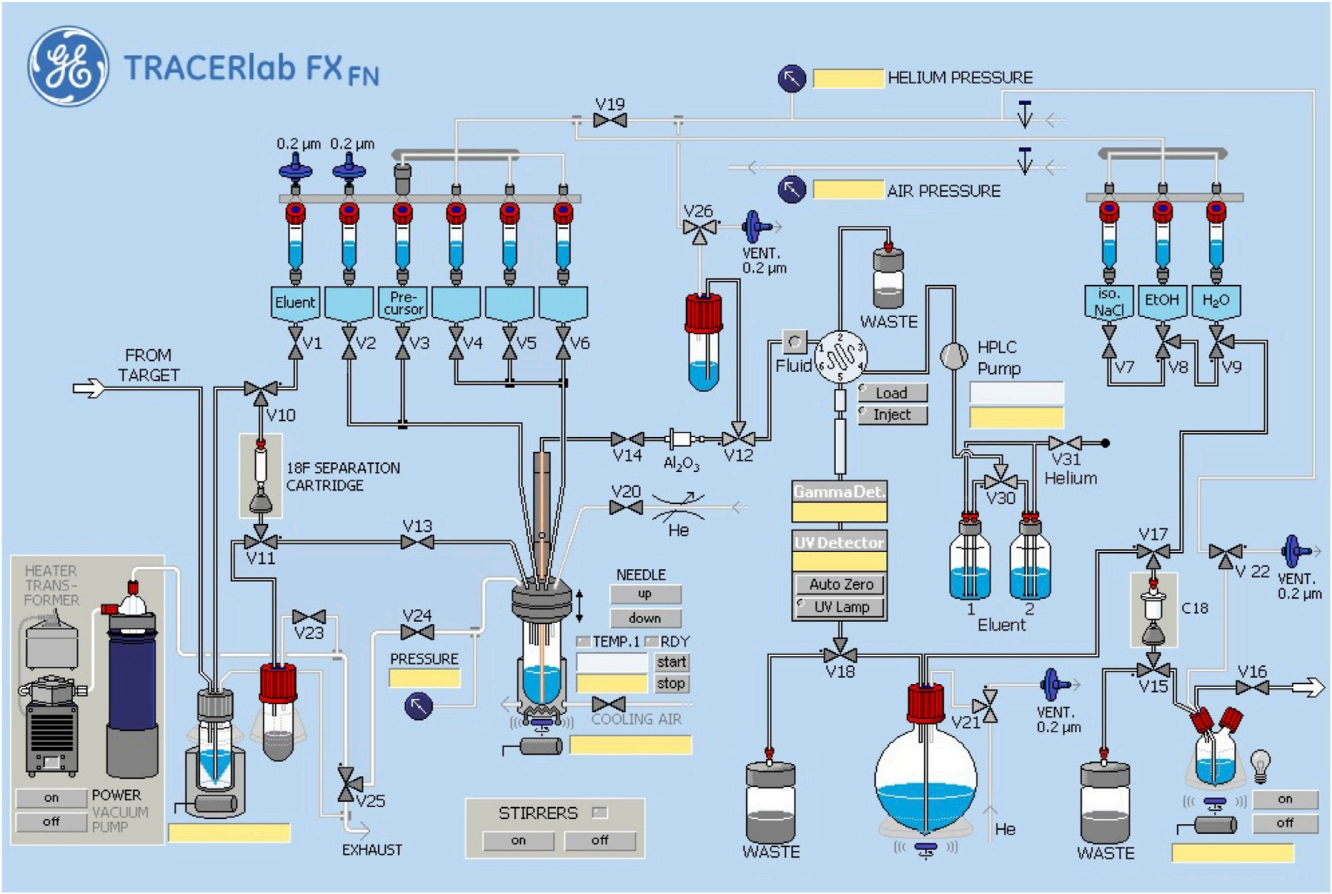
Synthesis module configuration.

[^18^F]fluoride was prepared using an automated GE TRACERLab FX_FN_ synthesis module. The TRACERLab was configured as shown in Figure 3 and the reagent vials were loaded as follows: Vial 1: potassium carbonate (3.5 mg in 0.5 ml water); Vial 2: kryptofix-2.2.2 (15 mg in 1.0 ml ethanol); Vial 3: precursor **15** (5.0 mg in 1,000 μL DMSO); Vial 6: HPLC buffer (50% ethanol, 50 mM NH_4_OAc, 0.2% acetic acid, pH 4.73, 3.5 ml); Vial 7: 0.9% sodium chloride for injection, USP (4.5 ml); Vial 8: ethanol (0.5 ml); and Vial 9: sterile water for injection, USP (10 ml); round bottom flask: Milli-Q water (60 ml); product vial: 0.9% sodium chloride for injection, USP (5.0 ml).

Fluorine-18 was produced *via* the ^18^O(p,n)^18^F nuclear reaction using a GE PET Trace cyclotron equipped with a high yield fluorine-18 target at 55 µA to produce 74 GBq (2 Ci) of fluorine-18. The [^18^F]fluoride was delivered from the cyclotron [in a 2.5 ml bolus of (^18^O)H_2_O] and trapped on a QMA-light sep-pak (preconditioned with sodium bicarbonate) to remove (^18^O)H_2_O. (^18^F)fluoride was then eluted into the reaction vessel using aqueous potassium carbonate (3.5 mg in 0.5 ml of water). A solution of kryptofix-2.2.2 (15 mg in 1.0 ml of ethanol) was then added to the reaction vessel and the [^18^F]fluoride was dried by azeotropic evaporation of the water-ethanol mixture.

Azeotropic drying was achieved by heating the reaction vessel to 100°C under vacuum for 4 min and a flow of argon for 5 min. The reactor was then cooled to 60°C and resultant fluoride was dried with a stream of He. Precursor **15** (5 mg) in anhydrous DMSO (1 ml) was added and the labeling reaction heated at 80°C for 10 min with stirring. Subsequently, the reaction mixture was cooled from 80 to 50°C, and quenched by addition of HPLC buffer (3.5 ml). The product was purified by semi-preparative HPLC (column: Luna PFP(2), 250 × 10 mm-10 μ; mobile phase: 50% ethanol, 50 mM NH_4_OAc, 0.2% AcOH, pH 4.73; flow rate = 3 ml/min; UV = 254 nm, Supplementary Figure S4 for a typical semi-preparative HPLC trace). The product peak (∼15–18 min retention time) was collected for maximum 2 min and diluted into a round-bottom flask containing 60 ml of Milli-Q water. The solution was then passed through a C18 Sep-Pak to trap the product on the C18 cartridge. The C18 cartridge was washed with 10 ml of sterile water. The product was eluted with 0.5 ml of ethanol, and diluted with 9.5 ml of saline. The final formulation was passed through a 0.22 μm filter into a sterile dose vial and submitted for QC testing using standard methods [Supplementary Material and previous reports (Scott and Kilbourn, 2007; Shao et al., 2014)]. Analytical HPLC of the formulated dose confirmed high radiochemical purity (Supplementary Figure S5), while coinjection with unlabeled reference standard **1** confirmed radiochemical identity (Supplementary Figure S6).

#### 2.1.3 Modifications to Include Ascorbic Acid

The radiosynthesis was conducted as described above, but with the following modifications: 1) a round-bottom flask contained 60 ml of Milli-Q water and 100 µL of ascorbic acid, USP (500 mg/ml); and 2) the final dose was formulated in 5% ethanol in saline, with addition of 100 µL of ascorbic acid, USP (500 mg/ml). Analysis of residual kryptofix in batches containing ascorbic acid used the modified TLC spot test (Scott and Kilbourn, 2007).

## 3 Results and Discussion

**FIGURE 4:**
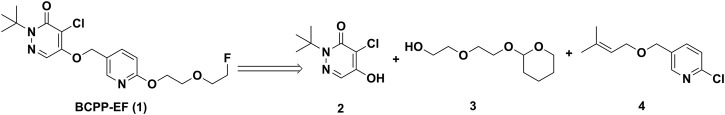
Proposed retrosynthetic strategy for the synthesis of 2-(*tert*-butyl)-4-chloro-5-((6-(2-(2-fluoroethoxy)ethoxy)pyridin-3-yl)methoxy)pyridazin-3(2*H*)-one (BCPP-EF) **(1)**.

**FIGURE 5:**
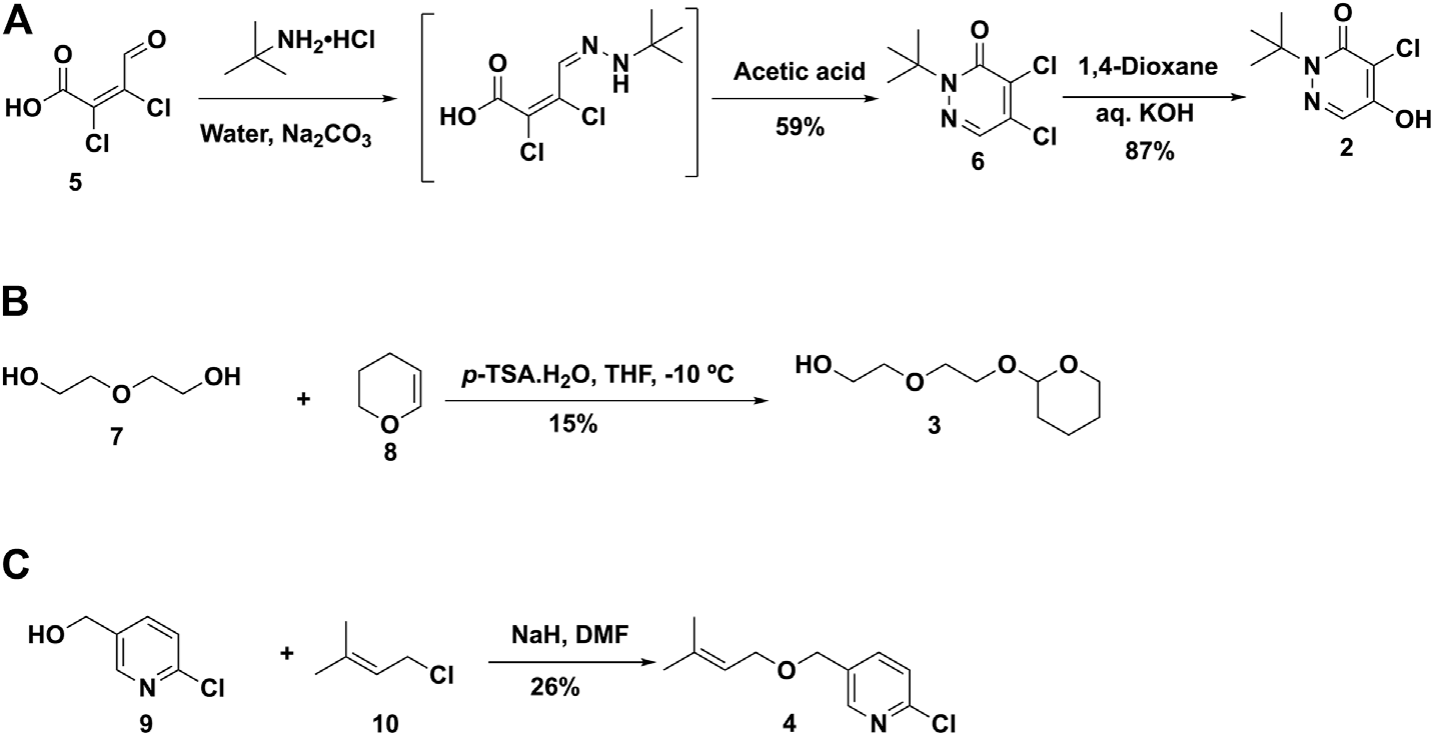
Synthesis of fragment **2 (A), 3 (B)** and **4 (C)** for the synthesis of BCPP-EF.

3.1.1. Chemistry. Based on previous reports (Nishiyama et al., 2014; Tsukada et al., 2018), the synthetic plan for BCPP-EF (**1**) was laid out, but steps were modified as needed to improve the synthesis (Harada et al., 2013). The retrosynthetic analysis of **1** resulted in three major fragments **2**, **3**, and **4** (Figure 4). Following literature reports, the synthesis of fragment **2** was achieved by treating mucochloric acid with *tert*-butylamine.hydrochloride in water with sodium carbonate (Harada et al., 2013). The generated hydrazone intermediate was subjected to acetic acid reflux conditions and resulted in dichloropyridazine analogue **6** in 59% yield. The intermediate **6**, under basic hydrolysis, resulted in partial hydrolyzed fragment **2** in 87% yield (Figure 5A).

Following literature reports, we started to build fragment **3** by treatment of diethylene glycol with dihydropyran using *p*-toluenesulfonic acid monohydrate as an acid catalyst at −10°C in 15% yield (Figure 5B). Next, considering the synthesis of fragment **4**, reaction conditions were modified from the reported route to have an improved and safe procedure (Harada et al., 2013). 6-Chloropyridin-3-yl-methanol **9** was treated with NaH to generate the alkoxide anion at room temperature. After evolution of H_2_ stopped, a solution of 1-chloro-3-methyl-2-butene (**10**) in DMF was added into the reaction mixture. The reaction was heated at 50°C for 24 h and purified to isolate the desired fragment **4** in 26% yield (Figure 5C).

**FIGURE 6:**
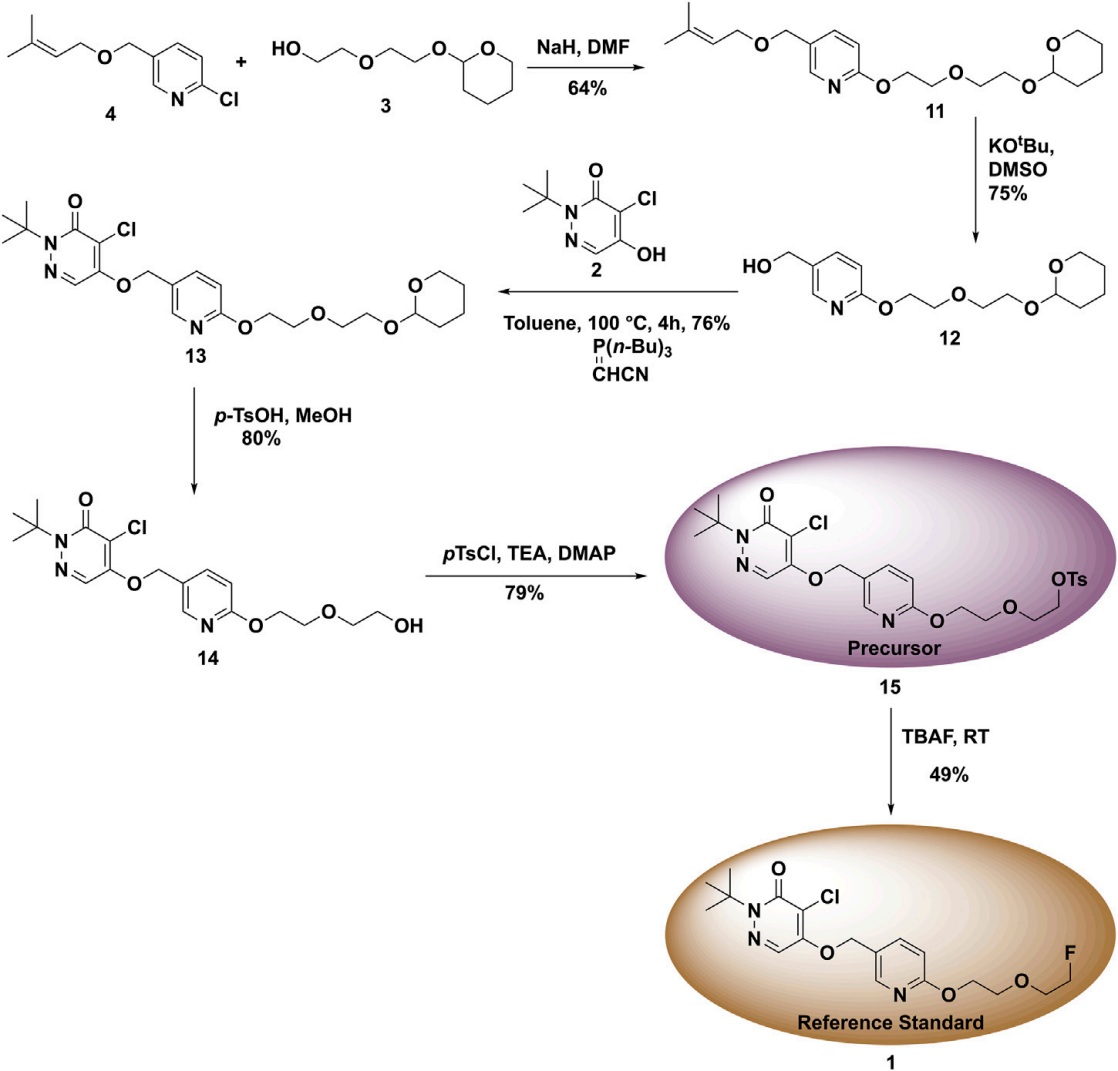
Synthesis of reference standard **1** and precursor **15** for used in the radiosynthesis of **(** ^**18**^ **F)1**.

With fragments **2**, **3**, and **4** in hand, we started the synthesis of labeling precursor **15** and unlabeled BCPP-EF reference standard **1**. In the original report (Harada et al., 2013), the coupling of **3** and **4** was conducted under microwave conditions. However, to make this synthesis more accessible we investigated the coupling of **3** and **4** under conventional heating conditions. To begin with, fragments **3** and **4** were dissolved in DMF and NaH was added. The reaction mixture was originally heated at 60°C for 6 h which resulted in desired product **11**, albeit at low (26%) yield (Figure 6). In an effort to improve the yield of the **11,** we increased the reaction temperature from 60 to 100°C and extended the reaction time to 12 h. To our delight, this resulted in an increase in the yield of **11** from 26 to 64%. Next, we deprotected the 1-chloro-3-methyl-2-butyl group from **11** with potassium *tert*-butoxide in DMSO at 60°C for 40 min, generating deprotected alkyl alcohol **12** at 75% yield. In literature precedents, we found that coupling of **12** with fragment **2** was carried out using Mitsunobu conditions. Unfortunately, in our hands the Mitsunobu conditions provided a low yield of **13**, and the product was difficult to purify. In previous work on a similar scaffold we had employed the Tsunoda reagent (cyanomethylenetributylphosphorane) (Kaur et al., 2021), and were gratified to observe that treatment of **12** with fragment **2** and the Tsunoda reagent generated intermediate **13** at 76% yield.

Next, the THP group of protected product **13** was removed under acidic conditions using p-toluenesulfonic acid hydrate in methanol. This generated the desired deprotected product **14** at 80% yield. To access the radiolabeling precursor, the hydroxyl group of intermediate **14** was tosylated with *p*-toluene sulfonyl chloride in DCM which generated **15** at 79% yield (Figure 6). Finally, to synthesize unlabeled BCPP-EF reference standard **1**, the tosylate **15** was reacted with TBAF at room temperature to give **1** at 49% yield.

3.1.2. Radiochemistry. To date there are no reports regarding an automated synthesis of BCPP-EF using a commercial radiochemistry synthesis module. To facilitate clinical studies under good manufacturing practice (GMP) we needed to develop an automated synthesis of (^18^F)BCPP-EF. We began by dissolving precursor **15** (10 mg) in anhydrous acetonitrile (1,000 μL) and reacted with azeotropically dried (^18^F)fluoride at 80°C for 10 min (Table 1, entry 1) as had been previously described. The crude reaction mixture was purified by semi-preparative HPLC using a perfluorophenyl-capped matrix column (Luna-PFP(2), Phenomenex) with an acetonitrile-based mobile phase (40% acetonitrile, 20 mM NH_4_OAc, 0.2% acetic acid, 4 ml/min). **(** ^**18**^ **F)1** (t_R_ = 12–15 min) was isolated at 46.3% RCY and at RCP of 100%.

**TABLE 1:** Conditions for the radiosynthesis of (^18^F)1.

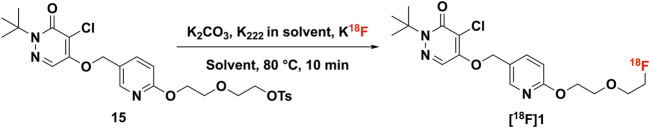					
**Entry**	**K**_**2**_ **CO**_**3**_	**K222**	**Precursor/solvent**	**RCY**	**RCP (%)**
1	3.5 mg in 0.5 ml water	15 mg K_222_ in 1 ml MeCN	10 mg/1,000 μL of MeCN	46.3% (*n* = 1)	100
2	3.5 mg in 0.5 ml water	15 mg K_222_ in 1 ml EtOH	10 mg/1,000 μL of DMSO	24.3% (*n* = 2)	97
3	3.5 mg in 0.5 ml water	15 mg K_222_ in 1 ml EtOH	5 mg/1,000 μL of DMSO	21.6% (*n* = 5)	98

As we considered clinical use of [^18^F]BCPP-EF further, we were curious if a green radiosynthesis could be developed in line with our previous work in this area (Shao et al., 2014; Stewart et al., 2015). The underlying premise of green (radio)chemistry is the design of products and processes that minimize or eliminate the use and generation of hazardous substances (U.S. EPA, 2021). In the context of fluorine-18 radiochemistry, this mainly involves replacing more toxic class 2 solvents (e.g., MeCN) with less toxic class 3 solvents (e.g., ethanol, DMSO). An additional benefit is that for radiosyntheses employing only class 3 solvents, residual solvent analysis does not need to be conducted on every batch but instead can be reduced to an annual quality control test (Shao et al., 2014; Stewart et al., 2015). In order to avoid the use of class 2 solvents in the synthesis of [^18^F]BCPP-EF, we investigated whether acetonitrile (injection limit in humans 4.1 mg/day) could be replaced with ethanol (50 mg/day) for azeotropic drying of [^18^F]fluoride and in the HPLC mobile phase, and with DMSO (50 mg/day) for the reaction solvent. We replaced K_222_ in acetonitrile (15 mg/ml in MeCN) for azeotropic drying with K_222_ in ethanol (15 mg/ml in EtOH) as we have done in prior work (Stewart et al., 2015), since both the MeCN/H_2_O and EtOH/H_2_O azeotropes have similar boiling points and are interchangeable in our experience. Secondly, the reaction solvent was changed from MeCN to DMSO, another common solvent for ^18^F-fluorination reactions (Table 1, entry 2). Lastly, the crude reaction mixture was once more purified using a perfluorophenyl-capped matrix semipreparative column (Luna-PFP(2), Phenomenex) but instead using an ethanolic mobile phase (50% ethanol, 50 mM NH_4_OAc, 0.2% acetic acid, 3 ml/min). To our delight, the (^18^F)BCPP-EF was obtained in 24.3% yield (Table 1, Entry 2) and in 97% RCP. It was found that reducing the amount of precursor from 10 to 5 mg did not decrease the yield of the desired product to a significant extent (Table 1, entry 2 vs. Table 1, entry 3). Thus a fully automated synthesis using 5 mg of precursor provided 15.99 ± 3.09 GBq (432.2 ± 83.6 mCi) of **[** ^**18**^ **F]1** in 21.6% decay-corrected RCY [*n* = 4, based upon 2.0 Ci of starting [^18^F]fluoride], >98% radiochemical purity and high molar activity [242.4 ± 105.7 GBq/μmol (6,521 ± 2,843 Ci/mmol)].

For reformulation, the purified **[** ^**18**^ **F]1** was trapped on a C18 (Waters, 1 cc vac) cartridge, and the cartridge was rinsed with water to remove the residual HPLC solvent/buffers. Different conditions were explored to find the best conditions for elution. We compared elution of the product with ethanol (0.5 ml), vs. ethanol/tween 80 with or without ascorbic acid (Table 2). All of the formulation conditions worked well to elute the product, did not show any evidence of radiolytic decomposition and maintained radiochemical purity of greater than 95% after 5 h. In our hands, there was no appreciable benefit to inclusion of tween 80, used in the original synthesis (Harada et al., 2013). Moreover, the formation of white precipitate was observed in ethanol/tween 80/ascorbic acid formulated dose after 2 h (Table 2, Entry 3). As such, we omitted the use of Tween 80 to simplify the final formulation as it was not required.

**TABLE 2:** Reformulation conditions for the [^18^F]1.

Entry	Reformulation conditions	Activity used (mCi)	Recovered in vial (mCi)	% Recovered
1	Ethanol (0.5 ml)	11.91	10.94	91.8
2	Ethanol (0.66 ml) + Tween 80 (0.16 ml)	11.21	10.01	89.3
3	Ethanol (0.5 ml) + Tween 80 (0.1 ml) + Ascorbic Acid (0.1 ml)	8.32	8.04	96.6

Lastly, encouraged by these results, we validated the synthesis of **[** ^**18**^ **F]1** to confirm suitability for production of batches for clinical use. Three process verifications runs were completed and produced [^18^F]**1** at 154 ± 31 mCi activity yield [7.7 ± 1.6% non-decay corrected radiochemical yield based upon 2.0 Ci of [^18^F]fluoride], >95% radiochemical purity and molar activity of 6,801 ± 2,154 Ci/μmol (251,637 ± 79,726 GBq/μmol) (Table 3). Synthesis time was 61–65 min from EOB. Quality control testing of [^18^F]BCPP-EF **1** was conducted according to the guidelines outlined in Chapter <823> of the US Pharmacopeia, and previously reported QC procedures (Supplementary Material). As shown in Table 3, batches 1–3 met all acceptance criteria confirming suitability for future clinical imaging studies. HPLC analysis confirmed batches were stable for at least 1 h which is sufficient for our needs. However, we found evidence of slow radiolysis such that by 3 h post-end-of-synthesis, RCP had dropped to 87%. To remedy this issue, we conducted a fourth validation run in which ascorbic acid was included in the final formulation. This inclusion allowed extension of the product shelf life to at least 8 h.

**TABLE 3:** Quality control data of [^18^F]BCPP-EF validation radiosyntheses.

QC test	Acceptance criteria	Batch 1 result	Batch 2 result	Batch 3 result	Batch 4 result^a^
Radiochemical Purity	≥90%	>99%	95%	96%	>99%
Radioactive Strength	NLT 10 mCi/10 ml @EOS	109 mCi/10 ml	175 mCi/10 ml	170 mCi/10 ml	163 mCi/10 ml
Active Ingredient Concentration	Report Results (μg/ml)	0.67 μg/ml	0.80 μg/ml	0.83 μg/ml	1.67 μg/ml
Molar Activity	Report Results (Ci/mmol)	6,460 mCi/µmol	8,696 mCi/µmol	8,147 mCi/µmol	3,901 mCi/µmol
pH	4.5–7.5	5.0	5.0	5.0	5.5
Visual Inspection	Clear, colourless, no ppt	Clear, colourless, no ppt	Clear, colourless, no ppt	Clear, colourless, no ppt	Clear, colourless, no ppt
Radiochemical Identity (HPLC)	RRT: 0.9–1.1	1.04	1.02	1.01	1.01
Radionuclide Identity	105–115 min	107.5 min	106.7 min	110.3 min	109.4 min
Residual Kryptofix	<50 μg/ml	<50 μg/ml	<50 μg/ml	<50 μg/ml	<50 μg/ml
Filter membrane Integrity	≥44 psi	51 psi	51 psi	52 psi	52 psi
Bacterial Endotoxin	≤17.5 EU/mL	<2.00 EU/mL	<2.00 EU/mL	<2.00 EU/mL	<2.00 EU/mL

Post-Release QC test	Release Criteria	Batch 1 Result	Batch 2 Result	Batch 3 Result	Batch 4 Result
1 h stability	≥90%	99%	94%	96%	97%
3 h	≥90%	n.d	n.d	87%^b^	98%
8 h	≥90%	n.d	n.d	n.d	97%^b^
Sterility	Sterile (no microbial growth)	Sterile	Sterile	Sterile	Sterile

a Ascorbic acid was added to the formulated product.

b Without ascorbic acid, shelf life = 1 h, with ascorbate, shelf life = 8 h; n.d. = not determined.

## 4 Conclusion

A fully automated radiosynthesis of [^18^F]BCPP-EF for PET imaging of MC-1 was developed. The highlights of the current method are its straightforward chemistry, simplicity, good radiochemical yields, and compatibility with commercially available radiochemistry synthesis modules for automated production of the radioligand at excellent radiochemical purity and high molar activity. Validation batches confirmed that batches of [^18^F]BCPP-EF produced using this method are suitable for use in clinical PET studies.

## Data Availability

The original contributions presented in the study are included in the article/Supplementary Material, further inquiries can be directed to the corresponding author.
