# Supplementary material for: Automated Synthesis of 18F-BCPP-EF {2-tert-Butyl-4-Chloro-5-{6-[2-(2[18F]fluoroethoxy)-Ethoxy]-Pyridin-3-ylmethoxy}-2H-Pyridazin-3-One for Imaging of Mitochondrial Complex 1 in Parkinson’s Disease
Source: Front Chem. 2022 Mar 30;10:878835. doi: 10.3389/fchem.2022.878835 (PMC9005973; doi:10.3389/fchem.2022.878835)
Supplement: Supplementary file 1 [file DataSheet1.docx]

Supplementary Material

Automated Synthesis of ^18^F-BCPP-EF {2-*tert*-Butyl-4-chloro-5-{6-[2-(2[^18^F]fluoroethoxy)-ethoxy]-pyridin-3-ylmethoxy}-2*H*-pyridazin-3-one for Imaging of Mitochondrial Complex 1 in Parkinson’s Disease

**Table of Contents**

1. Chemistry and Characterization…………………………………………………………................2
   1.1. General Considerations
   1.2. Synthesis of Intermediate 2, 3 and 4

1.3. Synthesis of Precursor 15 and Standard 1

1. Radiochemistry…………………………………………………………………………………….6
   2.1. General Considerations.
   2.2. Procedure for Radiochemical Synthesis of 2-(*tert*-Butyl)-4-chloro-5-((6-(2-(2-(fluoro-^18^*F*) ethoxy)ethoxy)pyridin-3-yl)methoxy) pyridazin-3(2*H*)-one [^18^F]1

1. ^1^H NMR, ^13^C NMR and HRMS Spectra………………………………………………………….11
   3.1. 2-(*tert*-Butyl)-4,5-dichloropyridazin-3(2*H*)-one (6)
   3.2. 2-(*tert*-Butyl)-4-chloro-5-hydroxypyridazin-3(2*H*)-one (2)
   3.3. 2-(2-((Tetrahydro-2*H*-pyran-2-yl)oxy)ethoxy)ethan-1-ol (3)

3.4. 2-Chloro-5-(((3-methylbut-2-en-1-yl)oxy)methyl)pyridine (4)

3.5. 5-(((3-Methylbut-2-en-1-yl)oxy)methyl)-2-(2-(2-((tetrahydro-2*H*-pyran-2-yl)oxy)ethoxy) ethoxy) pyridine (11)

3.6. (6-(2-(2-((Tetrahydro-2*H*-pyran-2-yl)oxy)ethoxy)ethoxy)pyridin-3-yl)methanol (12)

3.7. 2-(*tert*-Butyl)-4-chloro-5-((6-(2-(2-((tetrahydro-2*H*-pyran-2-yl)oxy)ethoxy)ethoxy)pyridin-3-yl) methoxy)pyridazin-3(2*H*)-one (13)

3.8. 2-(*tert*-Butyl)-4-chloro-5-((6-(2-(2-hydroxyethoxy)ethoxy)pyridin-3-yl)methoxy)pyridazin-3 (2*H*)-one (14)

3.9. 2-(2-((5-(((1-(*tert*-Butyl)-5-chloro-6-oxo-1,6-dihydropyridazin-4-yl)oxy)methyl)pyridin-2-yl) oxy)ethoxy)ethyl4-methylbenzenesulfonate (15)

3.10. 2-(*tert*-Butyl)-4-chloro-5-((6-(2-(2-fluoroethoxy)ethoxy)pyridin-3-yl)methoxy)pyridazin-3(2*H*)-one (1)

1. **Chemistry and Characterization**
   1. **General Considerations**

Unless otherwise stated all the chemicals were purchased from commercial suppliers and used without purification. Automated flash chromatography was performed with a Biotage Isolera Prime system. High-performance liquid chromatography (HPLC) was performed using a Shimadzu LC-2010A HT system equipped with a Bioscan B-FC-1000 radiation detector. ^1^H and ^13^C NMR spectra were collected on a Varian 500 NMR (500 MHz for ^1^H NMR and 125 MHz for ^13^C NMR), in Methanol-d_4_ or CDCl_3_ unless otherwise indicated, δ in ppm relative to tetramethylsilane (δ = 0), *J* in Hz. Mass spectra were measured on an Agilent Q-TOF HPLC-MS.

- 1. Synthesis of intermediate **2**, **3** and **4**

**Supplement Figure 1:** Synthesis of intermediate **2**, **3** and **4**

**2-(*tert*-Butyl)-4,5-dichloropyridazin-3(2*H*)-one (6):** Mucochloric acid **5** (5.0 g, 29.6 mmol) was dissolved in H_2_O (44 mL) and Na_2_CO_3_ (1.53 g, 14.43 mmol) was added to the solution. The reaction mixture was cooled at 0 °C in a water ice-bath for 15 min. Next, *tert*-Butylamine hydrochloride (3.7 g, 29.6 mmol) was introduced into the reaction mixture, and the resulting mixture was stirred for 2.5 h. The generated precipitate was collected on sintered glass filter and washed with cold H_2_O and dried by pulling with vacuum. The dried material was re-dissolved in acetic acid (50 mL) and refluxed for an hour. The resultant mixture was cooled to room temperature, and then acetic acid was evaporated by rotary evaporator. The resulting residue was diluted with water and extracted with DCM (3 x 50 mL). The combined DCM layers were washed with water and dried over Na_2_SO_4_. The crude product was purified by silica gel flash chromatography using a hexane:EA gradient mobile system (10-20% EA/hexane), and **6** was collected as a white solid (3.85 g, 59%). ^1^H NMR (500 MHz; CDCl_3_)/δ (ppm): 7.72 (s, 1H), 1.64 (d, 9H).^13^C NMR (125 MHz; CDCl_3_)/δ (ppm): 156.8, 135.8, 134.7, 133.4, 66.9, 27.6. MS: Calculated for [M + H]^+^ (M = C_8_H_10_Cl_2_N_2_O) = 221.0248, actual *m/z* = 221.0187.

**2-(*tert*-Butyl)-4-chloro-5-hydroxypyridazin-3(2*H*)-one (2):** 2-(*tert*-Butyl)-4,5-dichloropyridazin-3(2*H*)-one **6** (3.85 g, 17.5 mmol) was dissolved in dioxane (20 mL). Aqueous KOH solution (2.88 g, 51.4 mmol in 28.8 mL) was prepared and was added to the solution. The resultant reaction mixture was refluxed overnight. The generated precipitate was filtered and washed with water/hexane. The pure product, **2**, was isolated as a white solid (3.1 g, 87%). ^1^H NMR (500 MHz; CD_3_OD)/δ (ppm): 7.70 (s, 1H), 1.64 (s, 9H).^13^C NMR (125 MHz; CD_3_OD)/δ (ppm): 160.0, 154.5, 129.4, 65.7, 27.0. MS: Calculated for [M + H]^+^ (M = C_8_H_11_ClN_2_O) = 203.0587, actual *m/z* = 203.0532.

**2-(2-((Tetrahydro-2*H*-pyran-2-yl)oxy)ethoxy)ethan-1-ol (3):** Diethylene glycol **7** (2.28 mL, 24 mmol) was dissolved in THF (4.0 mL). 3,4-Dihydropyran **8** (2.17 mL, 24 mmol) was dissolved in DCM (40 mL) and the resulting solution was added to the solution of **7**. The reaction mixture was cooled to -10 °C and *p*-TsOH.H_2_O (457 mg, 0.1 mmol) was added. It was stirred at -10 °C for an hour. The reaction mixture was quenched with brine and extracted with DCM (3 x 50 mL). The combined organic layers were rinsed with brine and dried over Na_2_SO_4._ The crude reaction mixture was purified by silica gel flash chromatography using a hexane:EA mobile phase gradient (40-80% EA/hexane). The purified product, **3**, was collected as a colorless oil (687 mg, 15% yield). R*_f_* = 0.23 (50% EA/hexane). ^1^H NMR (500 MHz; CDCl_3_)/δ (ppm): 4.67 – 4.56 (m, 1H), 3.86 (dtd, *J* = 17.5, 10.0, 8.4, 4.6 Hz, 2H), 3.79 – 3.54 (m, 7H), 3.49 (ddq, *J* = 20.6, 11.4, 5.2 Hz, 1H), 1.87 – 1.65 (m, 2H), 1.64 – 1.40 (m, 4H).^13^C NMR (125 MHz; CDCl_3_)/δ (ppm): 99.1, 98.9, 72.5, 72.4, 70.5, 70.4, 66.9, 66.7, 62.4, 62.2, 61.7, 30.5, 25.5, 25.5, 19.5, 19.4. HRMS: Calculated for [M + Na]^+^ (M = C_9_H_18_O_4_) = 213.1097, actual *m/z* = 213.1107.

**2-Chloro-5-(((3-methylbut-2-en-1-yl)oxy)methyl)pyridine (4):** (6-chloropyridin-3-yl)methanol **9** (2.5 g, 17.4 mmol) was dissolved in anhydrous DMF (15 mL) and NaH (1.51 g, 62.5 mmol) was added slowly in small portions (~200 mg) to the solution. The reaction mixture was stirred at room temperature for 30 min. 1-Chloro-3-methyl-2-butene **10** (7.0 mL, 61.9 mmol) was added to the reaction mixture, and the resulting mixture was heated at 50 °C for 24 h. Upon completion of the reaction, the mixture was cooled to room temperature, and quenched with *sat.* aqueous NH_4_Cl (5 mL). The quenched filtrate was extracted with EA (3 x 50 mL), washed with H_2_O and dried over Na_2_SO_4._ The crude reaction mixture was purified by silica gel flash chromatography using a hexane:EA gradient mobile phase (5-20% EA/hexane). The pure product, **4,** was collected as a yellow oil (966.3 mg, 26%). R*_f_* = 0.60 (50% EA/hexane). ^1^H NMR (500 MHz; CDCl_3_)/δ (ppm): 8.23 (d, *J* = 2.4 Hz, 1H), 7.56 (dd, *J* = 8.2, 2.4 Hz, 1H), 7.19 (d, *J* = 8.2 Hz, 1H), 5.32-5.23 (m, 1H), 4.37 (s, 2H), 3.93 (d, *J* = 7.0 Hz, 2H), 1.65 (s, 3H), 1.56 (s, 3H).^13^C NMR (125 MHz; CDCl_3_)/δ (ppm): 150.4, 148.8, 138.2, 137.7, 137.7, 133.1, 123.9, 121.4, 120.5, 68.3, 66.9, 66.3, 25.7, 18.0. HRMS: Calculated for [M + H]^+^ (M = C_11_H_14_ClNO) = 212.0842, actual *m/z* = 212.0841.

- 1. Synthesis of Precursor **15** and Standard **1**

**Supplement Figure 2:** Synthesis of Precursor **15** and Standard **1**

**5-(((3-Methylbut-2-en-1-yl)oxy)methyl)-2-(2-(2-((tetrahydro-2*H*-pyran-2-yl)oxy)ethoxy)ethoxy) pyridine (11):** 2-(2-((tetrahydro-2*H*-pyran-2-yl)oxy)ethoxy)ethan-1-ol **3** (1.0 g, 5.26 mmol) was dissolved in DMF (10 mL) and NaH (252 mg, 6.3 mmol) was added. After 30 minutes of stirring at room temperature, 2-chloro-5-(((3-methylbut-2-en-1-yl)oxy)methyl)pyridine **4** (1.11 g, 5.26 mmol) was added to the reaction mixture. The reaction mixture was heated at 100 °C for overnight. The reaction mixture was quenched with *sat.* NH_4_Cl (5 mL) and extracted with EA (3 x 50 mL). The combined organic layers were rinsed with water/brine and dried over Na_2_SO_4._ The crude reaction mixture was purified by silica gel flash chromatography using a hexane:EA mobile phase gradient (20-50% EA/hexane). The purified product **11** was collected as a colorless oil (1.23 g, 64%). R*_f_* = 0.66 (50% EA/hexane). ^1^H NMR (500 MHz; CDCl_3_)/δ (ppm): 7.95 (d, *J* = 2.4 Hz, 1H), 7.47 (dd, *J* = 8.5, 2.4 Hz, 1H), 6.65 (d, *J* = 8.5 Hz, 1H), 5.25 (t, *J* = 6.9 Hz, 1H), 4.53 (t, *J* = 3.6 Hz, 1H), 4.39 – 4.34 (m, 2H), 4.28 (s, 2H), 3.86 (d, *J* = 7.0 Hz, 2H), 3.80 – 3.70 (m, 4H), 3.64 – 3.60 (m, 2H), 3.53 – 3.48 (m, 1H), 3.41 – 3.34 (m, 1H) 1.76 – 1.33 (m, 12H),^13^C NMR (125 MHz; CDCl_3_)/δ (ppm): 163.8, 146.2, 139.0, 137.4, 125.4, 120.8, 111.1, 98.8, 70.5, 70.5, 69.6, 69.1, 66.6, 66.4, 65.2, 62.1, 30.5, 25.7, 25.4, 19.4, 18.0. HRMS: Calculated for [M + H]^+^ (M = C_20_H_31_NO_5_) = 366.2280, actual *m/z* = 366.2288.

**(6-(2-(2-((Tetrahydro-2*H*-pyran-2-yl)oxy)ethoxy)ethoxy)pyridin-3-yl)methanol (12):** 5-(((3-methylbut-2-en-1-yl)oxy)methyl)-2-(2-(2-((tetrahydro-2*H*-pyran-2-yl)oxy)ethoxy)ethoxy) pyridine **11** (571 mg, 1.56 mmol) was dissolved in DMSO (15 mL) and potassium *tert*-butoxide (1.75 g, 15.64 mmol) was added. The reaction mixture was heated at 60 °C for 40 min. The reaction mixture was quenched with *sat.* aqueous NH_4_Cl (5 mL) and extracted with EA (3 x 50 mL). The combined organic layers were rinsed with brine/water and dried over Na_2_SO_4._ The crude reaction mixture was purified by silica gel flash chromatography using a hexane:EA mobile phase gradient (50% EA/hexane-100% EA). The purified product, **12**, was isolated as a yellow oil (350 mg, 75%). R*_f_* = 0.30 (50% EA/hexane). ^1^H NMR (500 MHz; CDCl_3_)/δ (ppm): 8.05 (d, *J* = 2.4 Hz, 1H), 7.60 (dd, *J* = 8.5, 2.4 Hz, 1H), 6.76 (d, *J* = 8.4 Hz, 1H), 4.60 (d, *J* = 14.5 Hz, 3H), 4.45 (t, *J* = 4.9 Hz, 2H), 3.90 – 3.80 (m, 5H), 3.71 (t, *J* = 4.9 Hz, 2H), 3.66 – 3.57 (m, 1H), 3.54 – 3.45 (m, 1H), 1.84 – 1.75 (m, 1H), 1.72 – 1.66 (m, 1H), 1.61 – 1.46 (m, 4H).^13^C NMR (125 MHz; CDCl_3_)/δ (ppm): 163.3, 145.4, 138.5, 129.2, 111.3, 98.9, 70.6, 69.7, 66.7, 65.3, 62.4, 62.2, 30.5, 25.4, 19.4. HRMS: Calculated for [M + K]^+^ (M = C_15_H_23_NO_5_) = 336.1213, actual *m/z* = 336.1217.

**2-(*tert*-Butyl)-4-chloro-5-((6-(2-(2-((tetrahydro-2*H*-pyran-2-yl)oxy)ethoxy)ethoxy)pyridin-3-yl) methoxy)pyridazin-3(2*H*)-one (13):** (6-(2-(2-((tetrahydro-2*H*-pyran-2-yl)oxy)ethoxy)ethoxy)pyridin-3-yl)methanol **12** (200 mg, 1.1 mmol) and 2-(*tert*-Butyl)-4-chloro-5-hydroxypyridazin-3(2*H*)-one **2** (123 mg, 1.0 mmol) were dissolved in anhydrous toluene (5 mL). Cyanomethylenetributylphosphorane (240 μL, 1.5 mmol) was then added and the resultant reaction mixture was heated at 100 °C overnight. Upon completion of the reaction, toluene was evaporated using a rotary evaporator at reduced pressure. The crude reaction mixture was purified by silica gel flash chromatography using a hexane:EA mobile phase gradient (30%-60% EA/hexane). The purified product **13** was collected as a colorless oil (221 mg, 76%). R*_f_* = 0.50 (50% EA/hexane). ^1^H NMR (500 MHz; CDCl_3_)/δ (ppm): 8.17 (q, *J* = 2.0 Hz, 1H), 7.76 (s, 1H), 7.66 (dq, *J* = 8.6, 2.3 Hz, 1H), 6.83 (dt, *J* = 8.6, 2.3 Hz, 1H), 5.22 (s, 2H), 4.63 – 4.61 (m, 1H), 4.51 – 4.45 (m, 2H), 3.89 – 3.81 (m, 4H), 3.74 – 3.71 (m, 2H), 3.64 – 3.59 (m, 1H), 3.50 – 3.45 (m, 1H), 1.85 – 1.66 (m, 2H), 1.62 (s, 9H), 1.59 – 1.45 (m, 2H).^13^C NMR (125 MHz; CDCl_3_)/δ (ppm): 64.1, 159.0, 153.5, 146.1, 146.1, 138.6, 138.6, 125.1, 123.4, 118.6, 111.9, 111.9, 98.9, 70.6, 69.5, 69.5, 66.7, 66.5, 65.6, 65.6, 62.2, 30.5, 28.0, 27.8, 25.4, 19.4. HRMS: Calculated for [M + H]^+^ (M = C_23_H_32_ClN_3_O_6_) = 482.2068, actual *m/z* = 482.2063.

**2-(*tert*-Butyl)-4-chloro-5-((6-(2-(2-hydroxyethoxy)ethoxy)pyridin-3-yl)methoxy)pyridazin-3(2*H*)-one (14):** 2-(*tert*-Butyl)-4-chloro-5-((6-(2-(2-((tetrahydro-2*H*-pyran-2-yl)oxy)ethoxy)ethoxy)pyridin-3-yl) methoxy)pyridazin-3(2*H*)-one **13** (233 mg, 0.48 mmol) was dissolved in MeOH (5 mL) and *p*-TsOH.H_2_O (4.6 mg, 0.02 mmol) was added to the solution. The reaction mixture was stirred at room temperature for 16 h. Upon completion of the reaction, the mixture was filtered to remove solids and quenched with *sat.* aqueous NH_4_Cl (5 mL). The quenched filtrate was extracted with EA (3 x 50 mL), washed with H_2_O and dried over Na_2_SO_4._ The crude reaction mixture was purified with silica gel flash chromatography using a hexane:EA gradient mobile phase (50% EA/hexane-100% EA). Purified product **14** as a colorless oil collected (153 mg, 80% yield). R*_f_* = 0.28 (50% EA/hexane). ^1^H NMR (500 MHz; CD_3_OD)/δ (ppm): 8.23 (d, *J* = 2.4 Hz, 1H), 8.17 (s, 1H), 7.80 (dd, *J* = 8.6, 2.4 Hz, 1H), 6.86 (d, *J* = 8.6 Hz, 1H), 5.37 (s, 2H), 4.50 – 4.42 (m, 2H), 3.88 – 3.80 (m, 2H), 3.69 – 3.66 (m, 2H), 3.63 – 3.61 (m, 2H), 1.64 (s, 9H).^13^C NMR (125 MHz; CD_3_OD)/δ (ppm): 165.4, 160.9, 155.8, 147.9, 140.6, 127.6, 125.6, 118.3, 112.3, 73.7, 70.7, 70.5, 67.6, 66.7, 62.2, 28.2. HRMS: Calculated for [M + H]^+^ (M = C_18_H_24_ClN_3_O_5_) = 398.1483, actual *m/z* = 398.1485.

**2-(2-((5-(((1-(*tert*-Butyl)-5-chloro-6-oxo-1,6-dihydropyridazin-4-yl)oxy)methyl)pyridin-2-yl)oxy) ethoxy)ethyl4-methylbenzenesulfonate (15):** 2-(*tert*-Butyl)-4-chloro-5-((6-(2-(2-hydroxyethoxy)ethoxy)pyridin-3-yl)methoxy)pyridazin-3(2*H*)-one **14** (192 mg, 0.48 mmol) was dissolved in DCM (5 mL), and triethylamine (669 μL, 4.8 mmol) and catalytic DMAP (15 mg) were added. The reaction mixture was cooled to 0 °C. *p*-Toluenesulfonyl chloride (138.6 mg, 0.72 mmol) was added into the reaction mixture. The resultant reaction mixture was stirred at room temperature for 16 h. Upon completion of the reaction, the mixture was quenched with *sat.* aqueous NH_4_Cl (5 mL). The quenched mixture was extracted with EA (3 x 50 mL), washed with H_2_O and dried over Na_2_SO_4._ The crude reaction mixture was purified by silica gel flash chromatography using a hexane:EA gradient mobile phase (30-50% EA/hexane). The purified product was collected as a colorless oil (210 mg, 79%). R*_f_* = 0.60 (50% EA/hexane). ^1^H NMR (500 MHz; CD_3_OD)/δ (ppm): 8.13 (d, *J* =1.7 Hz, 1H), 7.77 (s, 1H), 7.72 (d, *J* = 8.6, 2H), 7.63 (dd, *J* = 8.6, 2.5 Hz, 1H), 7.26 – 7.22 (m, 2H), 6.75 (d, *J* = 8.6, 1H), 5.19 (s, 2H), 4.36 – 4.32 (m, 2H), 4.15 – 4.10 (m, 2H), 3.73 – 3.64 (m, 4H), 2.35 (s, 3H), 1.57 (s, 9H). ^13^C NMR (125 MHz; CD_3_OD)/δ (ppm): 171.0, 163.8, 158.9, 153.5, 146.2, 144.8, 138.6, 132.9, 129.8, 127.9, 125.1, 123.6, 118.3, 111.6, 69.6, 69.5, 69.2, 68.6, 66.4, 66.2, 65.2, 60.3, 27.8, 21.8, 21.5, 21.0, 14.1. HRMS: Calculated for [M + H]^+^ (M = C_25_H_30_ClN_3_O_7_S) = 552.1571, actual *m/z* = 552.1578.

**2-(*tert*-Butyl)-4-chloro-5-((6-(2-(2-fluoroethoxy)ethoxy)pyridin-3-yl)methoxy)pyridazin-3(2*H*)-one (1):** 2-(2-((5-(((1-(*tert*-Butyl)-5-chloro-6-oxo-1,6-dihydropyridazin-4-yl)oxy)methyl)pyridin-2-yl)oxy) ethoxy)ethyl4-methylbenzenesulfonate **15** (110 mg, 0.2 mmol) and TBAF (1.0 M in THF) (600 μL, 0.6 mmol) were added to a flask to give a solution. The reaction mixture was stirred at room temperature for 16 h. Upon completion of the reaction, the mixture was concentrated under vacuum. The crude reaction mixture was purified by silica gel flash chromatography using a hexane:EA gradient mobile phase (25-35% EA/hexane). The pure product was collected as a colorless oil (39.1 mg, 49%). R*_f_* = 0.66 (50% EA/hexane). ^1^H NMR (500 MHz; CDCl_3_)/δ (ppm): 8.17 (d, *J* = 2.4, 1H), 7.75 (s, 1H), 7.66 (dd, *J* = 8.6, 2.5 Hz, 1H), 6.84 (d, *J* = 8.6, 1H), 5.21 (s, 2H), 4.64 – 4.60 (m, 1H), 4.54 – 4.47 (m, 3H), 3.90 – 3.85 (m, 2H), 3.83 – 3.79 (m, 1H), 3.78 – 3.74 (m, 1H), 1.62 (s, 9H).^13^C NMR (125 MHz; CDCl_3_)/δ (ppm): 164.0, 159.6, 154.1, 146.1, 138.6, 125.7, 123.5, 118.6, 111.5, 83.8, 82.4, 70.5, 70.3, 69.8, 69.5, 66.5, 65.4, 29.2.^19^F NMR (470 MHz, CDCl_3_)/δ (ppm): -222.7, -222.8, -222.9, -222.9, -222.9, -223.0, -223.0, -223.0, -223.1. HRMS: Calculated for [M + H]^+^ (M = C_18_H_23_ClFN_3_O_4_) = 400.1439, actual *m/z* = 400.1451.

1. **Radiochemistry**

**2.1. General Considerations.**

Unless otherwise stated, reagents and solvents were commercially available and used without further purification: sodium chloride, 0.9% USP, and sterile water for injection, USP, were purchased from Hospira; ethanol was purchased from American Regent; HPLC grade acetonitrile was purchased from Fisher Scientific. Other synthesis components were obtained as follows: sterile filters were obtained from Millipore; sterile product vials were purchased from Hollister-Stier; C18 Sep-Paks were purchased from Waters Corporation. C18 Sep-Paks were flushed with 10 mL of ethanol followed by 10 mL of Milli-Q water prior to use.

**2.2. Procedure for Radiochemical Synthesis of [^18^F]1.**

[^18^F]Fluoride was prepared using an automated GE TRACERLab FX_FN_ synthesis module. The TRACERLab was configured as shown in Figure 1 and the reagent vials were loaded as follows: Vial 1: potassium carbonate (3.5 mg in 0.5 mL water); Vial 2: kryptofix-2.2.2 (15 mg in 1.0 mL ethanol); Vial 3: precursor (5.0 mg in 1000 μL DMSO); Vial 6: HPLC buffer (50% Ethanol, 50 mM NH_4_OAc, 0.2% acetic acid, pH 4.73, 3.5 mL); Vial 7: 0.9% sodium chloride for injection, USP (4.5 mL); Vial 8: ethanol (0.5 mL); and Vial 9: sterile water for injection, USP (10 mL); round bottom flask: Milli-Q water (60 mL); product vial: 0.9% sodium chloride for injection, USP (5.0 mL).

**
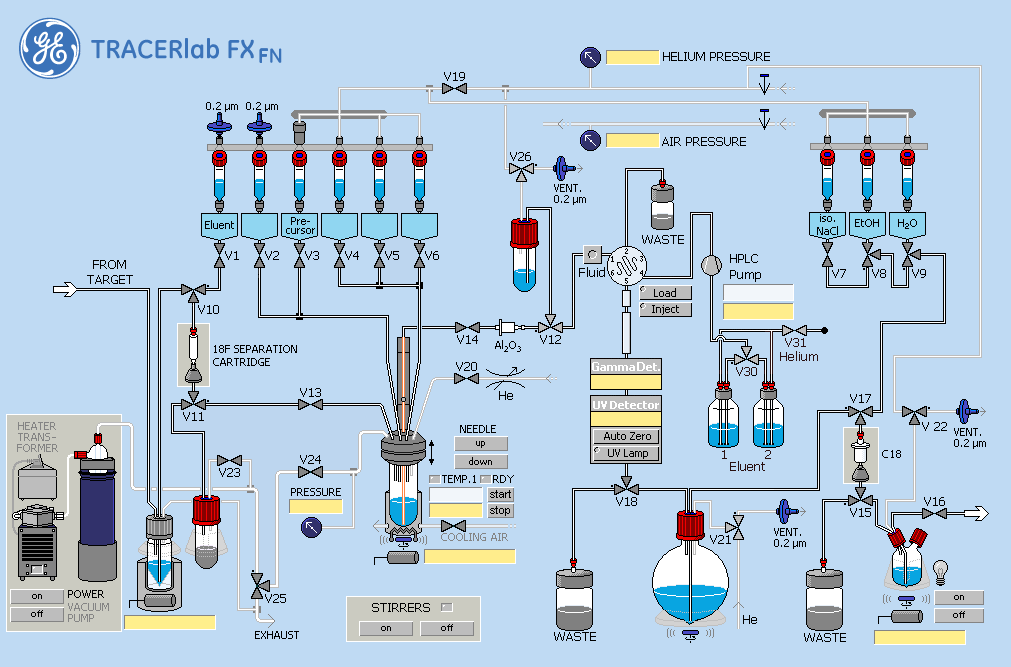
Supplement Figure 3:** Synthesis module configuration.

Fluorine-18 was produced *via* the ^18^O(p,n)^18^F nuclear reaction using a GE PET Trace cyclotron equipped with a high yield fluorine-18 target at 55 µA to produce 74 GBq (2 Ci) of fluorine-18.^1^ The [^18^F]Fluoride was delivered from the cyclotron (in a 2.5 ml bolus of [^18^O]H_2_O) and trapped on a QMA-Light Sep-Pak, which had been preconditioned with sodium bicarbonate, to remove [^18^O]H_2_O. [^18^F]Fluoride was then eluted into the reaction vessel using aqueous potassium carbonate (3.5 mg in 0.5 mL of water). A solution of kryptofix-2.2.2 (15 mg in 1.0 mL of ethanol) was then added to the reaction vessel and the [^18^F]fluoride was dried by azeotropic evaporation of the water-acetonitrile mixture. Evaporation was achieved by heating the reaction vessel to 100 °C under vacuum for 4 minutes and a flow of argon for 5 minutes. The reactor was then cooled to 60 °C and resultant fluoride was dried with a stream of He. The precursor (5 mg) in anhydrous DMSO (1 mL) was added and heated at 80 °C for 10 min with stirring. Subsequently, the reaction mixture was cooled from 80°C to 50°C, followed by quenching of reaction with the addition of HPLC buffer (3.5 mL). The reaction was loaded onto a semipreparative column (Luna PFP(2), 250 x 10 mm-10μ) and purified with using buffer (50% ethanol, 50 mM NH_4_OAc, 0.2% AcOH, pH 4.73, flow rate = 3 mL/min). The product peak (∼15-18 min retention time) was collected for maximum 2 minutes and diluted into a round-bottom flask containing 60 mL of Milli-Q water. The solution was then passed through a C18 Sep-Pak to trap the product on the C18 cartridge. The C18 cartridge was washed with 10 mL of sterile water. The product was eluted with 0.5 mL of ethanol, followed by 9.5 mL of saline. The final formulation was passed through a 0.2 μm sterile filter into a sterile dose vial. The final product was obtained in 15.99 ± 3.09 GBq (432.2 ± 83.6 mCi), 21.6% decay corrected yield, > 90% RCP, pH = 5-5.5, n = 4 in 61-65 minutes from the end of bombardment.


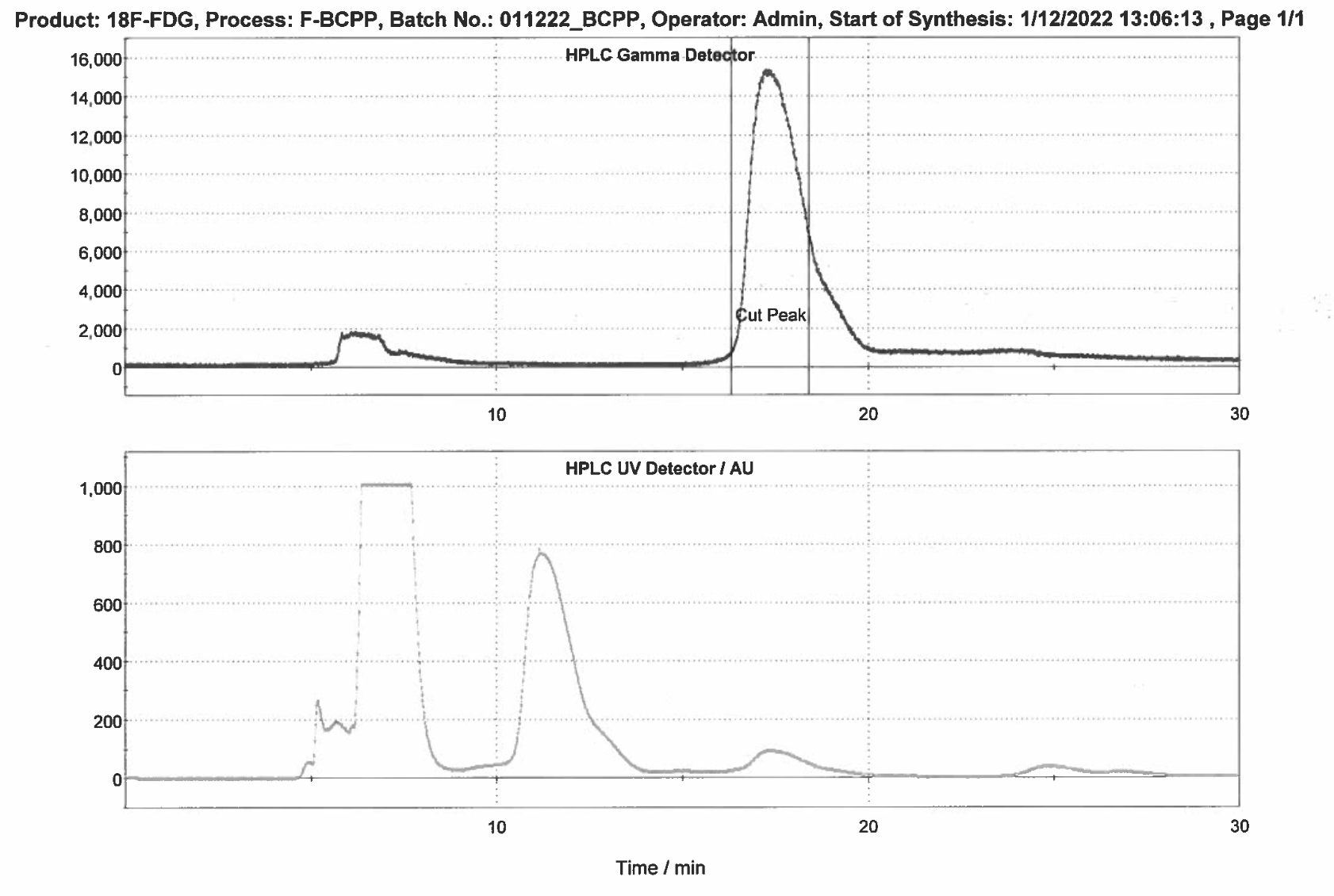


**Supplemental Figure 4:**

**Semi-preparative HPLC trace for [^18^F]1**: Column: Luna PFP, 250 x10 mm-10μ, flow rate: 3 mL/min, mobile phase: 50% Ethanol, 50 mM NH_4_OAc, 0.2% Acetic acid, pH 4.73, retention time: ~15-18 min.

**Note**: While we see tailing of the peak for **[^18^F]1** in the gamma trace (t_R_ ~15-18 min), this is unexplained but not due to the presence of a radiochemical impurity as we see no evidence of such in the associated analytical HPLC trace of the dose (see: Supplemental Figure 5).


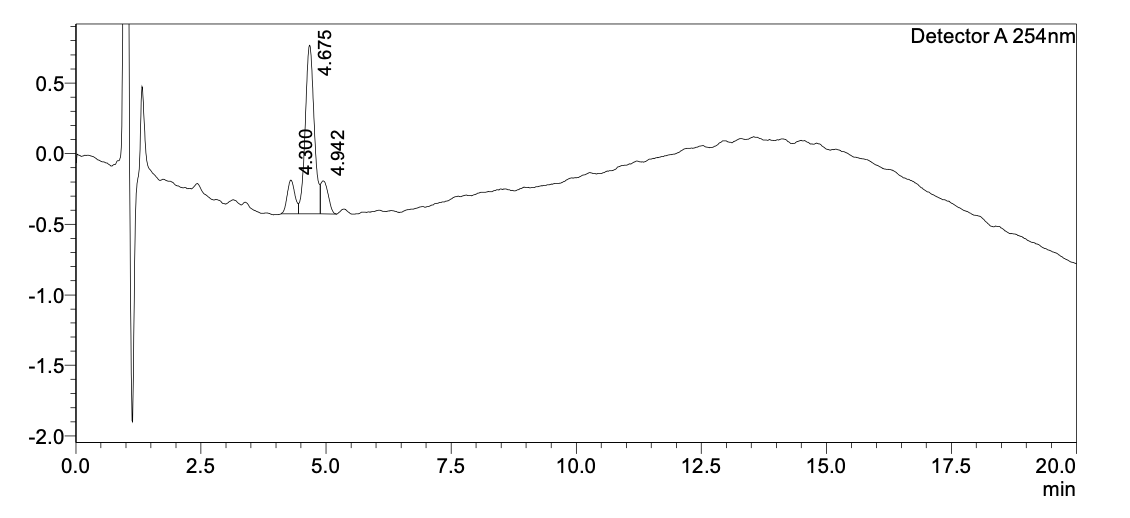

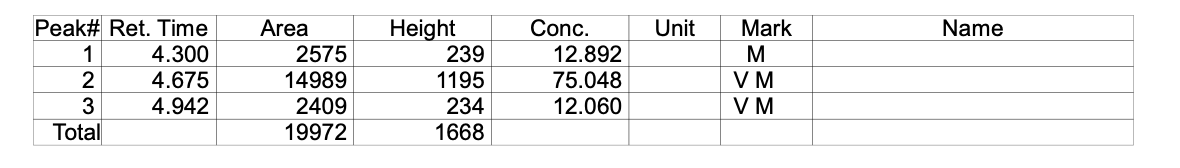

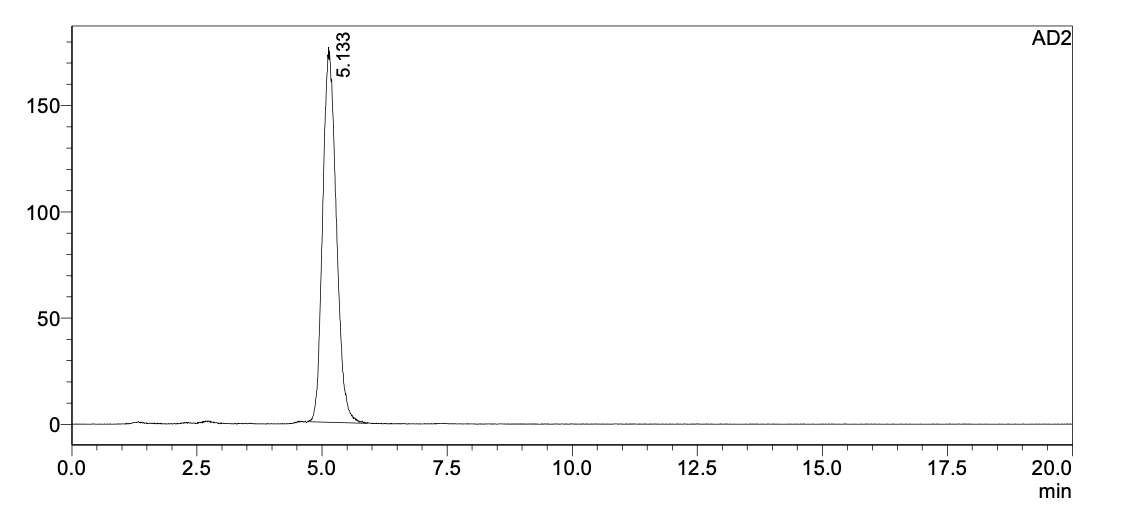

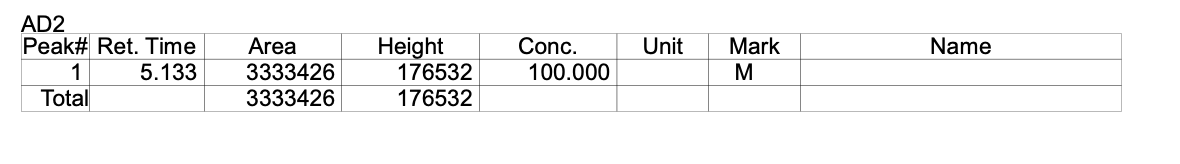


**Supplemental Figure 5:**

**Analytical HPLC trace for [^18^F]1**: Column: Luna PFP(2), 150x4.6 mm-5μ, flow rate: 2 mL/min, mobile phase: 40% Acetonitrile, 20 mM NH_4_OAc, 0.2% Acetic acid, pH 4.73, retention time: ~4-5 min.


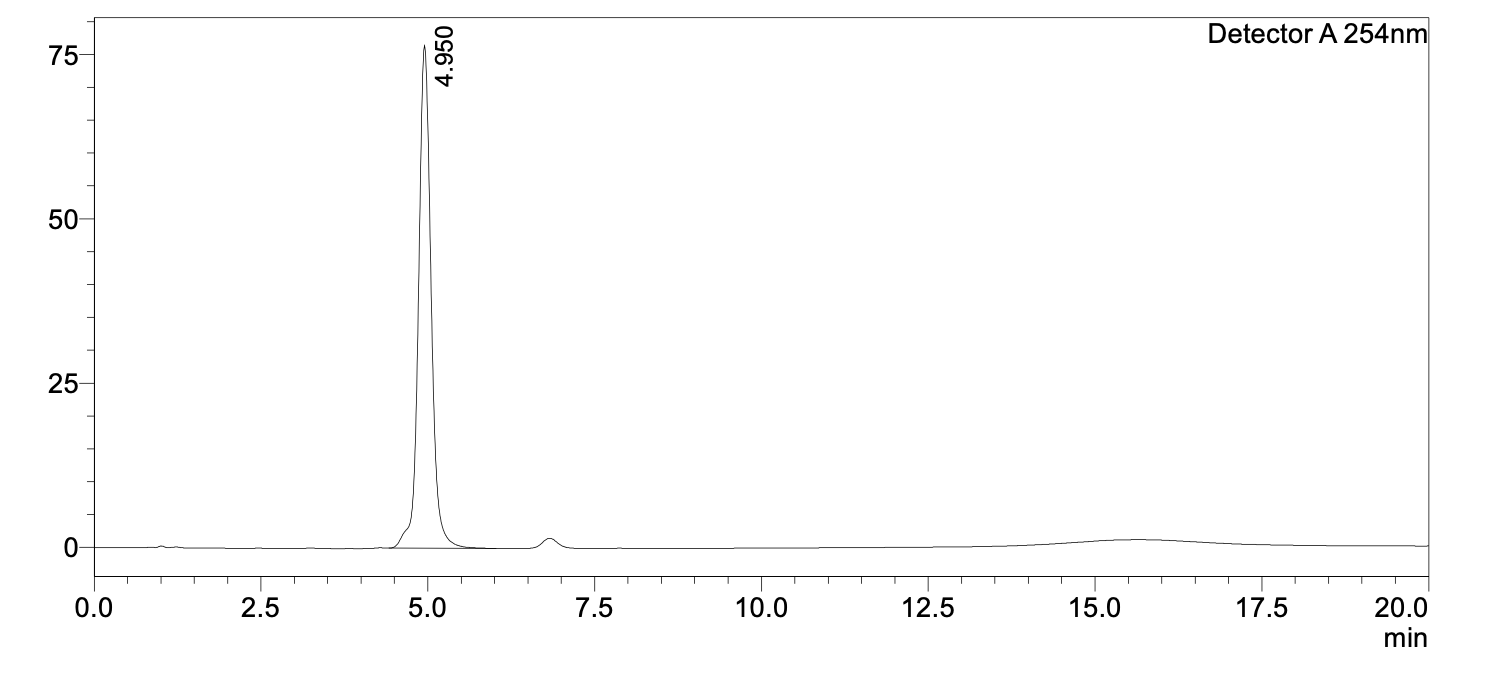

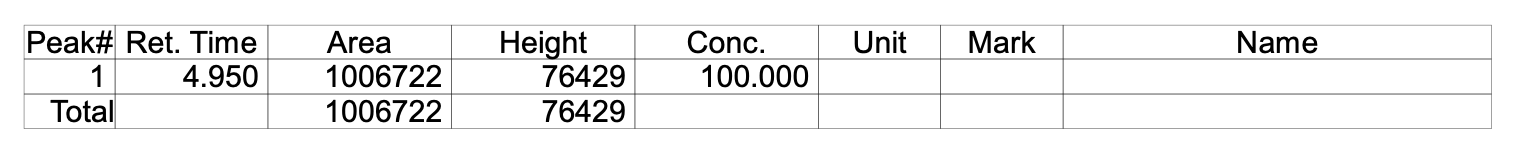

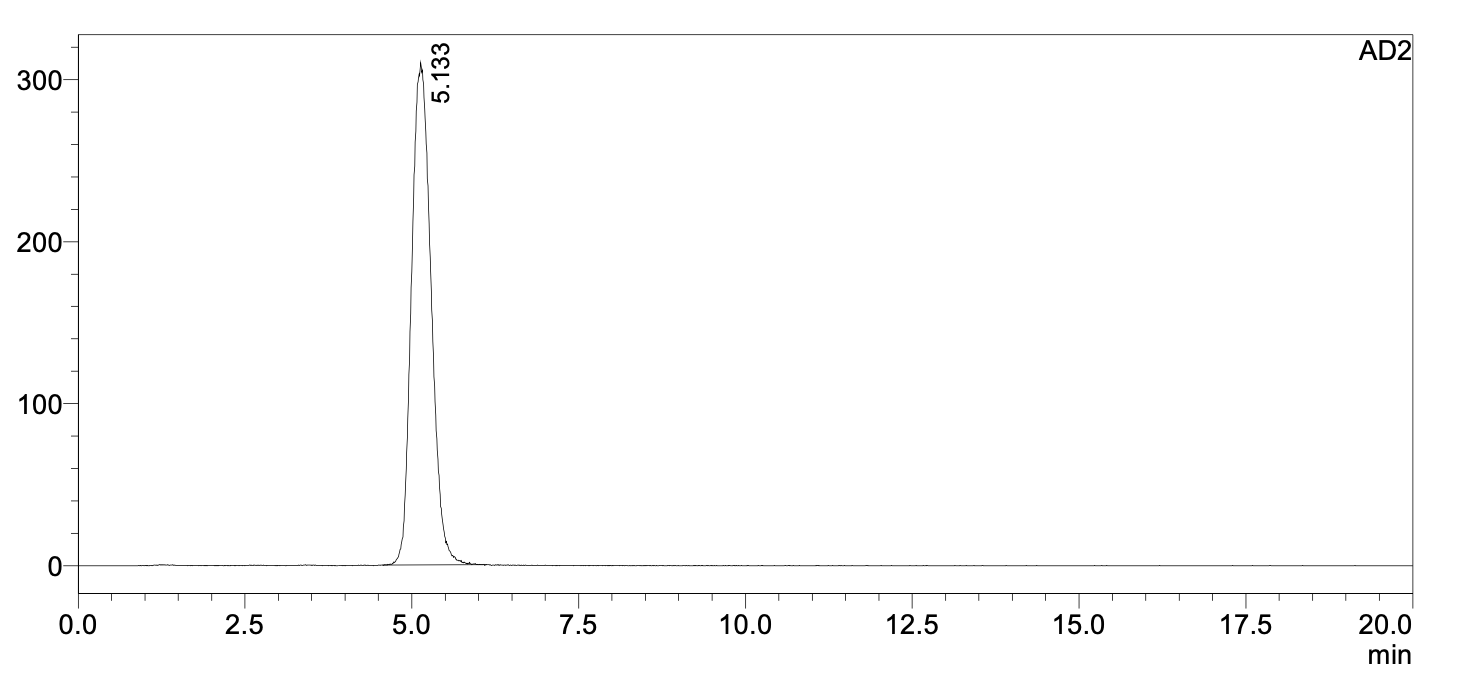

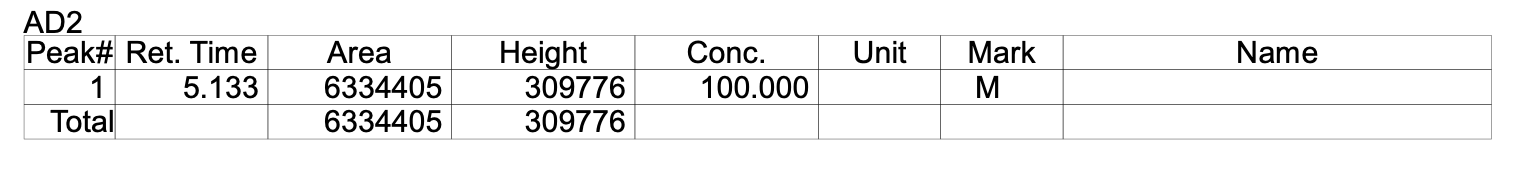


**Supplemental Figure 6:**

**Analytical HPLC trace for [^18^F]1**: Coinjection of standard compound **1** compound (UV) with purified reaction product from radiochemical synthesis. Purified radiochemical product injected with cold standard, HPLC method repeated from supplement Figure 7. Integrations provided.

1. **^1^H NMR, ^13^C NMR and HRMS Spectra**
   1. **2-(*tert*-Butyl)-4,5-dichloropyridazin-3(2*H*)-one (6)**

**^1^H NMR**

**Supplemental Figure 7**

**^13^C NMR**

**Supplemental Figure 8**

**LCMS**

**Mass predicted: 221 [M+H]^+^**

**Mass measured: 221 [M+H]^+^**

**M-butyl**

**Mass predicted: 221.0248 [M+H]^+^**

**Mass measured: 221.0187 [M+H]^+^**

**Supplemental Figure 9**

- 1. **2-(*tert*-Butyl)-4-chloro-5-hydroxypyridazin-3(2*H*)-one (2):**

**^1^H NMR**

**Supplemental Figure 10**

**^13^C NMR**

**Supplemental Figure 11**

**LCMS**

**Mass predicted: 203.0587 [M+H]^+^**

**Mass measured: 203.0532 [M+H]^+^**

**Supplemental Figure 12**

- 1. **2-(2-((Tetrahydro-2*H*-pyran-2-yl)oxy)ethoxy)ethan-1-ol (3):**

**^1^H NMR**

**Supplemental Figure 13**

**^13^C NMR**

**Supplemental Figure 14**

**HRMS**

**Mass predicted: 213.1097 [M+Na]^+^**

**Mass measured: 213.1107 [M+Na]^+^**

**Supplemental Figure 15**

- 1. **2-Chloro-5-(((3-methylbut-2-en-1-yl)oxy)methyl)pyridine (4):**

**^1^H NMR**

**Supplemental Figure 16**

**^13^C NMR**

**Supplemental Figure 17**

**HRMS**

**Mass predicted: 212.0841 [M+H]^+^**

**Mass measured: 212.0843 [M+H]^+^**

**Supplemental Figure 18**

- 1. **5-(((3-Methylbut-2-en-1-yl)oxy)methyl)-2-(2-(2-((tetrahydro-2*H*-pyran-2-yl)oxy)ethoxy)ethoxy) pyridine (11):**

**^1^H NMR**

**Supplemental Figure 19**

**^13^C NMR**

**Supplemental Figure 20**

**HRMS**

**Mass predicted: 366.2280 [M+H]^+^**

**Mass measured: 366.2288 [M+H]^+^**

**Supplemental Figure 21**

- 1. **(6-(2-(2-((Tetrahydro-2*H*-pyran-2-yl)oxy)ethoxy)ethoxy)pyridin-3-yl)methanol (12):**

**^1^H NMR**

**Supplemental Figure 22**

**^13^C NMR**

**Supplemental Figure 23**

**HRMS**

**Mass predicted: 336.1213 [M+K]^+^**

**Mass measured: 336.1217 [M+K]^+^**

**Supplemental Figure 24**

- 1. **2-(*tert*-Butyl)-4-chloro-5-((6-(2-(2-((tetrahydro-2*H*-pyran-2-yl)oxy)ethoxy) ethoxy) pyridin-3-yl)methoxy)pyridazin-3(2*H*)-one (13):**

**^1^H NMR**

**Supplemental Figure 25**

**^13^C NMR**

**Supplemental Figure 26**

**HRMS**

**Mass predicted: 482.2068 [M+H]^+^**

**Mass measured: 482.2063 [M+H]^+^**

**Supplemental Figure 27**

- 1. **2-(*tert*-Butyl)-4-chloro-5-((6-(2-(2-hydroxyethoxy)ethoxy)pyridin-3-yl)methoxy)pyridazin-3(2*H*)-one (14):**

**^1^H NMR**

**Supplemental Figure 28**

**^13^C NMR**

**Supplemental Figure 29**

**HRMS**

**Mass predicted: 398.1483[M+H]^+^**

**Mass measured: 398.1485 [M+H]^+^**

**Supplemental Figure 30**

- 1. **2-(2-((5-(((1-(*tert*-Butyl)-5-chloro-6-oxo-1,6-dihydropyridazin-4-yl)oxy)methyl)pyridin-2-yl)oxy)ethoxy)ethyl 4-methylbenzenesulfonate (15):**

**^1^H NMR**

**Supplemental Figure 31**

**^13^C NMR**

**Supplemental Figure 32**

**HRMS**

**Mass predicted: 552.1571 [M+H]^+^**

**Mass measured: 552.1578 [M+H]^+^**

**Supplemental Figure 33**

- 1. **2-(*tert*-Butyl)-4-chloro-5-((6-(2-(2-fluoroethoxy)ethoxy)pyridin-3-yl)methoxy)pyridazin-3(2*H*)-one (1):**

**^1^H NMR**

**Supplemental Figure 34**

**^13^C NMR**

**Supplemental Figure 35**

**^19^F NMR**

**Supplemental Figure 36**

**HRMS**

**Mass predicted: 400.1439 [M+H]^+^**

**Mass measured: 400.1420 [M+H]^+^**

**M+K**

**Supplemental Figure 37**
